# Canine Pyoderma and Otitis Externa: A Retrospective Analysis of Multidrug-Resistant Bacterial Carriage in Hong Kong

**DOI:** 10.3390/antibiotics14070685

**Published:** 2025-07-06

**Authors:** Wing Yu Chan, Stefan Hobi, Andrew Ferguson, Ibrahim Elsohaby

**Affiliations:** 1 Jockey Club College of Veterinary Medicine and Life Sciences, City University of Hong Kong, Hong Kong SAR 999077, China; wychan989-c@my.cityu.edu.hk; 2 Department of Veterinary Clinical Sciences, Jockey Club College of Veterinary Medicine and Life Sciences, City University of Hong Kong, Hong Kong SAR 999077, China; 3 CityU Veterinary Diagnostic Laboratory Co., Ltd., City University of Hong Kong, Hong Kong SAR 999077, China; andrew.ferguson@cityuvdl.com.hk; 4 Department of Infectious Diseases and Public Health, Jockey Club of Veterinary Medicine and Life Sciences, City University of Hong Kong, Hong Kong SAR 999077, China; 5 Centre for Applied One Health Research and Policy Advice (OHRP), City University of Hong Kong, Hong Kong SAR 999077, China

**Keywords:** antimicrobial susceptibility, MRSP, canine, multidrug resistance, otitis externa, pyoderma

## Abstract

**Background**: Canine pyoderma and otitis externa are prevalent bacterial skin infections in veterinary practice, frequently complicated by the emergence of multidrug-resistant (MDR) pathogens. **Objectives:** To investigate the frequency, antimicrobial resistance (AMR) profiles, and frequency of MDR bacterial isolates from dogs with pyoderma or otitis externa in Hong Kong. **Methods**: A retrospective study of bacterial isolates from 215 clinical samples collected from dogs presenting with pyoderma (*n* = 63) or otitis externa (*n* = 152) at veterinary clinics across Hong Kong between 2018 and 2022. Bacterial isolates were identified and subjected to antimicrobial susceptibility testing against 13 antimicrobial classes. **Results**: *Staphylococcus* spp., particularly *S. pseudintermedius*, were the most commonly isolated species, followed by *Pseudomonas* spp. and *Proteus* spp. High resistance rates were observed for orbifloxacin (61.3% in pyoderma; 76.7% in otitis externa), doxycycline (59.3%; 69.2%), clindamycin (62%; 68.9%), and enrofloxacin (50%; 55.5%). Most isolates were sensitive to ofloxacin, ticarcillin–clavulanate, tobramycin, ciprofloxacin, cefpodoxime, cefuroxime, and cefixime. MDR was detected in 67.5% of pyoderma and 66.8% of otitis externa isolates. Gram-negative bacteria exhibited significantly higher MDR rates than Gram-positive isolates. The multiple antibiotic resistance (MAR) index averaged 0.41 for pyoderma and 0.52 for otitis externa isolates. We found no significant associations between MDR and non-modifiable risk factors (i.e., age, sex, breed, and reproductive status). **Conclusions**: These findings highlight the critical need for prudent antimicrobial use and continuous surveillance of AMR trends in companion animals. A higher focus should be placed on topical antiseptic therapy, with oral antibiotics used only in exceptional cases and after susceptibility testing. From a One Health perspective, the potential transmission of MDR bacteria between companion animals and humans underscores the importance of a coordinated approach to antimicrobial stewardship across both veterinary and human medicine.

## 1. Introduction

Canine pyoderma, a frequently diagnosed dermatopathy in dogs, is characterized by a bacterial skin infection [1]. It is widely recognized as a secondary condition caused by underlying factors that disrupt the skin barrier or immune defenses. Among various conditions, hypersensitivities are the leading cause of pyoderma, contributing to 59% of the cases in affected dogs in a German study [2]. Based on anatomical location, pyoderma is classified as surface, superficial, or deep, affecting the epidermis, dermis, and subcutaneous tissue, respectively [3]. On the other hand, otitis externa is one of the most prevalent inflammatory conditions of the canine external auditory canal, with prevalence rates ranging from 5 to 20% [4,5]. Parasites, foreign bodies, endocrinopathies, and allergies, including food allergy and atopic dermatitis, are common primary causes of otitis externa, while bacteria and yeast often contribute as secondary factors. Other factors such as breed, humidity, excessive hair growth, and ear conformation can predispose animals to otitis externa by facilitating opportunistic bacterial colonization [6,7,8].

Many Gram-positive and Gram-negative bacteria have been associated with canine pyoderma and otitis externa [9]. Among these, *Staphylococcus* spp. are the predominant commensal bacteria on healthy animal skin but can act as opportunistic pathogens in pyoderma cases. *S. pseudintermedius*, *S. aureus*, and *S. schleiferi* are commonly identified as primary agents of canine pyoderma [10]. For instance, a study in Thailand found that *S. pseudintermedius* was responsible for 89% of staphylococcal pyoderma cases [11]. Other bacteria, such as *Proteus* spp., *Pseudomonas aeruginosa*, *E. coli*, and *Streptococcus* spp., may also contribute to the pathogenesis. Similarly, *S. pseudintermedius* is the most frequently reported bacterial pathogen in otitis externa, followed by *P. aeruginosa* [5,12]. Other commonly identified bacteria in otitis externa include *Streptococcus* spp., *Proteus* spp., *Corynebacterium* spp., and *E. coli* [3,12,13].

Topical therapy is considered the first-line approach to canine pyoderma and otitis externa. However, indiscriminate use of antimicrobials over the years has driven the emergence of multidrug-resistant (MDR; resistant to three or more classes of antimicrobial agents) *Staphylococcus* spp. [14]. A French study has reported that 42% of the *S. pseudintermedius* strains in pyoderma cases were MDR, meaning that they were simultaneously resistant to three or more antimicrobial classes [15]. Of particular concern are methicillin-resistant *S. pseudintermedius* (MRSP) and *S. aureus* (MRSA), which exhibit resistance to nearly all *β*-lactams, complicating the management of staphylococcal infections. Worryingly, these MRS strains often display resistance to additional antimicrobial classes, further narrowing treatment options [16].

Antimicrobial resistance (AMR) has emerged as a global threat to human and veterinary medicine. In Hong Kong, concerns about AMR are escalating [17]. A previous local study revealed a low rate of MRSP in healthy dogs [18]. However, research on MDR in canine pyoderma and otitis externa remains limited, creating a critical knowledge gap. Given the close interaction between humans and companion animals and the widespread use of broad-spectrum antimicrobials in veterinary practice [19], this study aims to (i) determine the frequency of common bacterial infections and their AMR profiles in clinical samples of canine pyoderma and otitis externa cases in Hong Kong between 2018 and 2022 and (ii) identify the frequency of MDR bacteria recovered from these cases. By addressing these objectives, this study seeks to provide veterinarians with evidence-based insights to optimize treatment strategies and mitigate the further development of AMR.

## 2. Results

### 2.1. Study Population

**Figure 1:**
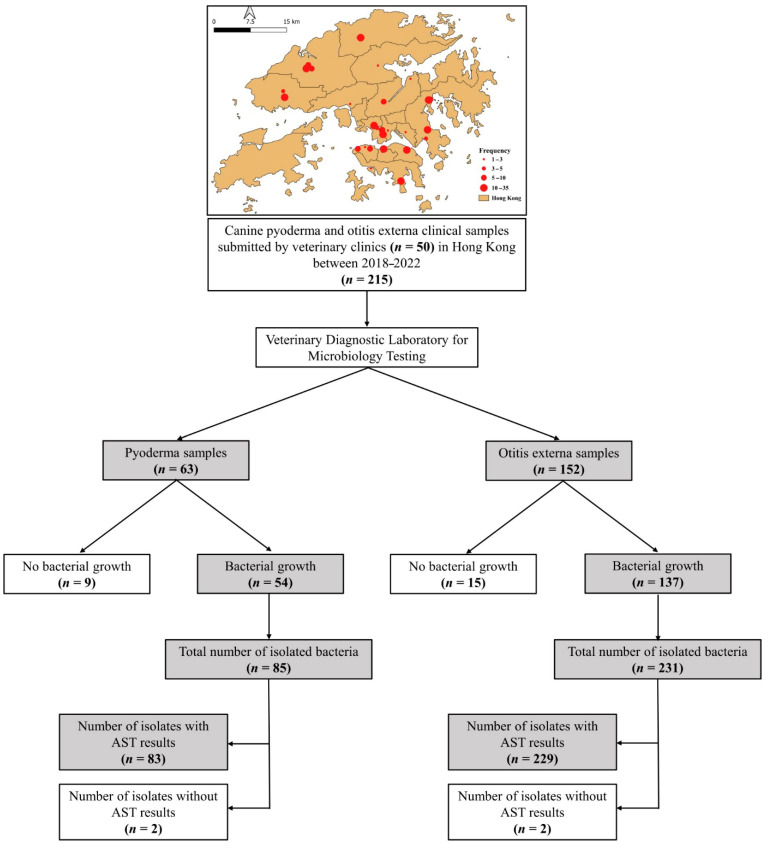
A schematic diagram illustrates the distribution of veterinary clinics that submitted canine pyoderma and otitis externa clinical samples to CityU VDL, along with a flowchart describing data extraction. AST = antimicrobial susceptibility test.

A total of 215 clinical samples were submitted to City University of Hong Kong Veterinary Diagnostic Laboratory (CityU VDL) between 2018 and 2022 by 50 veterinary clinics in Hong Kong for bacterial culture and antimicrobial susceptibility testing (AST) (Figure 1). Of these, 63 (29.3%) were from canine pyoderma cases and 152 (70.7%) from otitis externa cases.

For pyoderma cases, the age of the dogs ranged from 0.54 to 17.1 years, with a median of 6.8 years. Males accounted for 68.2% of cases, females 31.8%, and 44.4% of the dogs were desexed (Table 1). Most affected dogs were purebred (93.6%), with German Shepherd dogs being the most commonly represented breed (44.7%). Over half (65.1%) of the pyoderma samples were submitted in 2020 (33.3%) and 2022 (31.8%), and nearly 50% of the samples originated from the trunk region.

In otitis externa cases, dog ages ranged from 0.53 to 17.1 years, with a median of 8.1 years. The cohort consisted of 54.3% males, 45.7% females, and 68.4% desexed dogs. Almost all (98%) were purebred, with German Shepherd dogs (which have high moisture levels in their ear canals) again on the lead (69.3%), followed by Bernese Mountain dogs (11.69%). Of the otitis externa samples included in this study, 63.1% were submitted in 2020 (31.6%) and 2021 (28.8%), and 86.3% originated from a single ear (Table 1).

### 2.2. Microbiological Results

**Figure 2:**
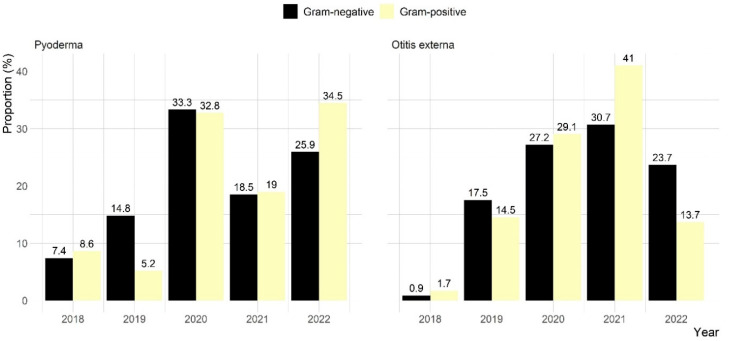
Distribution of Gram-positive and -negative bacterial isolates recovered from canine pyoderma and otitis externa clinical samples submitted to CityU VDL in Hong Kong over a five-year period.

**Figure 3:**
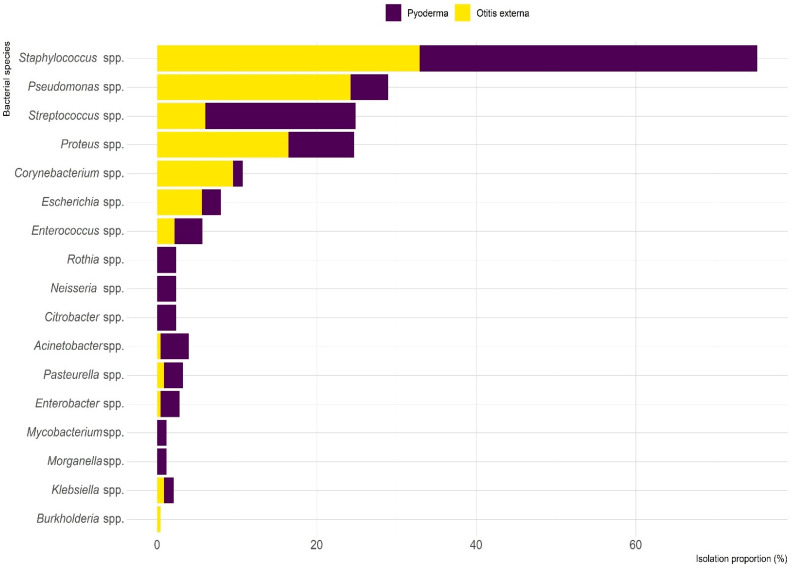
Frequency of bacterial isolates recovered from canine pyoderma and otitis externa clinical samples submitted to CityU VDL in Hong Kong over a five-year period.

Of the 63 clinical pyoderma samples, 54 (85.7%) showed bacterial growth, with 22 (35.2%) exhibiting single bacterial growth and 41 (64.8%) showing mixed growth. A total of 85 bacterial strains were isolated, representing 32 species. Of these, 58 (68.2%) were Gram-positive and 27 (31.8%) were Gram-negative (Figure 2). *Staphylococcus* spp. (42.4%) was the most commonly isolated genus, with 80.6% of *Staphylococcus* isolates identified as *S. pseudintermedius*. Other isolated staphylococcal species were *S. schleifer* (16.7%) and *S. aureus* (2.8%). Additional prevalent species in pyoderma cases were *Streptococcus* spp. (18.8%), *Proteus* spp. (8.2%), and *Pseudomonas* spp. (4.7%) (Figure 3).

Among the 152 otitis externa samples, 137 (90.1%) showed bacterial growth, with 79 (57.7%) displaying single growth and 58 (42.3%) mixed growth. A total of 231 isolates from 32 species were identified, with nearly equal representation of Gram-positive (50.6%) and Gram-negative (49.4%) bacteria (Figure 2). *Staphylococcus* spp. (32.9%) was the most frequently isolated genus, followed by *Pseudomonas* spp. (24.2%) and *Proteus* spp. (16.5%) (Figure 3).

### 2.3. Antimicrobial Susceptibility Testing

**Figure 4:**
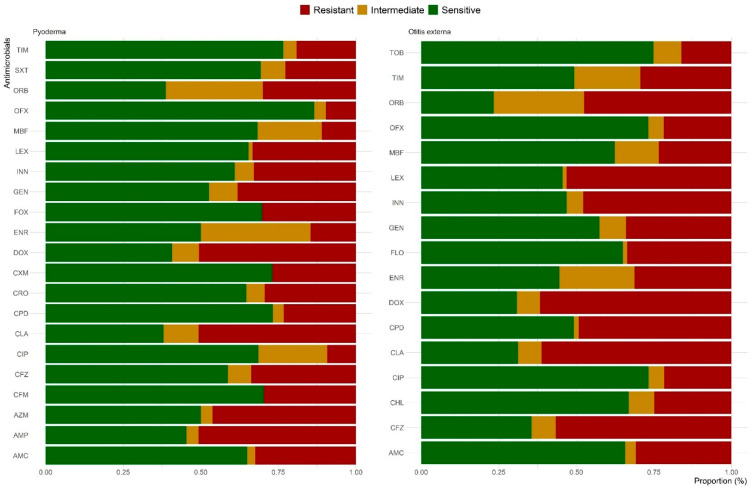
Frequency of antimicrobial resistance among bacterial isolates recovered from canine pyoderma and otitis externa clinical samples submitted to CityU VDL in Hong Kong over a five-year period. DOX, doxycycline; CLA, clindamycin; CFZ, cefazolin; LEX, cephalexin; CPD, cefpodoxime; INN, cefovecin; ORB, orbifloxacin; GEN, gentamicin; FLO, florfenicol; ENR, enrofloxacin; AMC, amoxicillin/clavulanate; TIM, ticarcillin–clavulanate; CHL, chloramphenicol; MBF, marbofloxacin; OFX, ofloxacin; CIP, ciprofloxacin; TOB, tobramycin; AMP, ampicillin; AZM, azithromycin; CFM, cefixime; CRO, ceftriaxone; CXM, cefuroxime; and SXT, trimethoprim–sulfamethoxazole.

The AST was performed on 83 of the 85 bacterial isolates recovered from pyoderma cases. The results showed that 94% of tested isolates were resistant to at least one antimicrobial (Figure 4). More than half of the isolates were resistant to clindamycin (62%), orbifloxacin (61.3%), doxycycline (59.3%), and ampicillin (54.7%), followed by azithromycin (50%) and enrofloxacin (50%). In contrast, 86.5%, 76.6%, 73.2%, and 73.1% of the isolates were sensitive to ofloxacin, ticarcillin–clavulanate, cefpodoxime, and cefuroxime, respectively. Among the predominant pyoderma species, more than half of *Staphylococcus* spp., 81.3% of *Streptococcus*, 85.7% of *Proteus* spp., and 100% of *Pseudomonas* spp. isolates were resistant to three or more antimicrobials (Table 2 and Figure S1).

Out of the 231 isolates recovered from otitis externa clinical samples, AST was performed on 229 isolates, with 93.9% of tested isolates showing resistance to at least one antimicrobial (Figure 4). High resistance rates were observed against orbifloxacin (76.7%), doxycycline (69.2%), clindamycin (68.9%), cefazolin (64.5%), enrofloxacin (55.5%), cephalexin (54.5%), and cefovecin (53.1%). However, two-thirds of the isolates were sensitive to tobramycin (74.9%), ciprofloxacin (73.3%), ofloxacin (73.2%), and chloramphenicol (66.9%). Among the predominant species, *Pseudomonas* spp. (100%), *Streptococcus* spp. (92.9%), and *Proteus* spp. (89.5%) isolates exhibited the highest rates of resistance to more than three antimicrobials (Table 2 and Figure S1).

### 2.4. MAR Index and MDR

**Figure 5:**
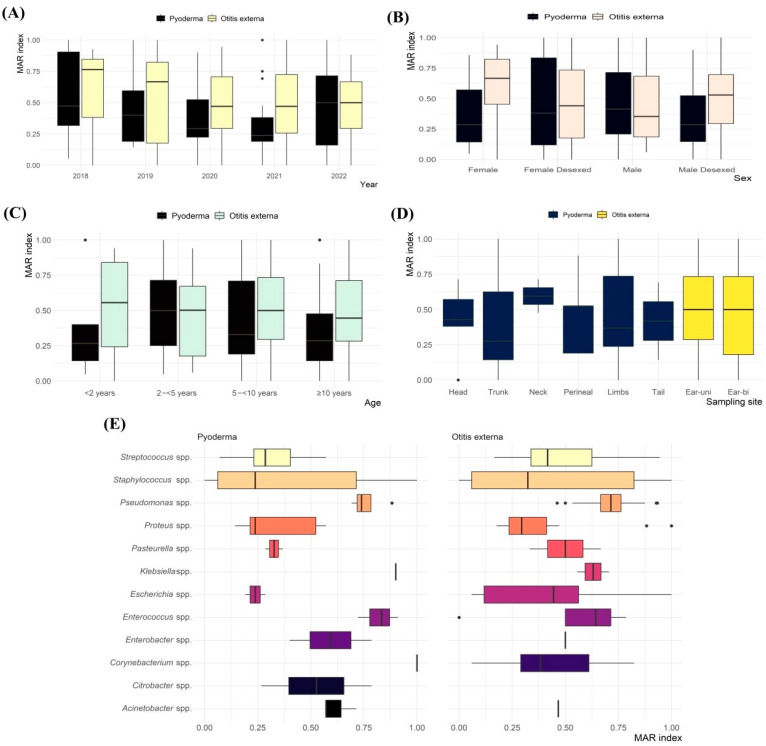
Multiple antimicrobial resistance (MAR) index for bacterial isolates recovered from canine pyoderma and otitis externa clinical samples submitted to CityU VDL in Hong Kong over a five-year period stratified by (**A**) sampling year; (**B**) sex; (**C**) age; (**D**) sampling site; and (**E**) bacterial genus.

The average MAR index of resistant isolates from pyoderma and otitis externa clinical samples was 0.41 and 0.52, respectively, ranging from 0.05 to 1.00 (Figure 5). Nearly 76% of the resistant isolates had an MAR index ≥ 0.2, indicating high levels of AMR.

No significant differences in the average MAR index were observed between isolates from pyoderma and otitis externa based on sampling year (Figure 5A), sex (Figure 5B), age (Figure 5C), or sampling site (Figure 5D). However, significant variation was found based on bacterial species (Figure 5E). Notably, comparisons such as *Streptococcus* spp. vs. *Pseudomonas* spp. (*p* = 0.002) and *Proteus* spp. vs. *Enterococcus* spp. (*p* = 0.016) from pyoderma, and *Proteus* spp. vs. *Pseudomonas* spp. (*p* = <0.001) from otitis externa showed distinct differences in the MAR index.

MDR was observed in 67.5% of isolates from pyoderma and 66.8% from otitis externa clinical samples. *Pseudomonas* spp., *Proteus* spp., *Enterococcus* spp., and *Streptococcus* spp. showed the highest rates of MDR (Table 2 and Figure S1). Gram-negative isolates exhibited significantly higher MDR levels than Gram-positive isolates (Table 3). In contrast, no significant differences in MDR frequency were found based on sampling year or site, age, sex, reproductive status, or breed.

## 3. Discussion

The present study investigated the frequency of bacterial infections and their AMR profiles in clinical samples of canine pyoderma and otitis externa cases submitted to CityU VDL by veterinary clinics in Hong Kong between 2018 and 2022. *Staphylococcus* spp., particularly *S. pseudintermedius*, were identified as the most prevalent bacterial genus isolated from both canine pyoderma (42.4%) and otitis externa (32.9%). These findings are consistent with numerous studies conducted across different geographical regions [10,12,20,21]. A recent meta-analysis by Tanveer et al. [22] further supports our results, reporting *Staphylococcus* spp. as the most frequently isolated genus (95.93%) in dogs with pyoderma and otitis externa in South Korea, with *S. pseudintermedius* (78.89%) being the predominant species. Similarly, studies from Spain [23], Italy [12], Serbia [5], and Tunisia [24] have also identified *S. pseudintermedius* as the most frequently isolated bacterium in canine dermatological infections.

The high proportion of *S. pseudintermedius* in our study (80.6% of staphylococcal pyoderma samples) is consistent with its recognized role as a resident organism of the canine skin microbiota that can become pathogenic under favorable conditions [25]. This observation aligns with the findings of Pinchbeck et al. [26], who reported that staphylococcal isolates from diseased skin are frequently clonally identical to commensal strains from healthy carriage sites, supporting the hypothesis that infections originate from endogenous rather than external sources. *S. pseudintermedius* possesses numerous virulence factors, including adhesins, toxins, and extracellular enzymes that enable the bacterium to effectively colonize host tissues, invade deeper layers, and evade the immune system [24]. The widespread occurrence of *S. pseudintermedius* across multiple geographical regions suggests that it has evolved specialized mechanisms to adapt to and thrive within the canine host environment. These characteristics make *S. pseudintermedius* a primary target for the development of therapeutic strategies and preventive measures in veterinary dermatology.

In addition to *Staphylococcus* spp., our study revealed *Pseudomonas* spp., particularly *P. aeruginosa*, as the second most common bacterial genus in otitis externa (24.2%) but less frequent in pyoderma (4.7%). This distribution aligns with findings reported in previous studies [5,27,28], which consistently identify *P. aeruginosa* as a clinically significant pathogen in canine otitis externa but less prevalent in skin infections. Arais et al. [27] specifically investigated *P. aeruginosa* in canine otitis externa and pyoderma in Brazil, emphasizing its clinical significance in ear infections. Similarly, Garcias et al. [28] reported that *Pseudomonas* spp. represented 20% of bacterial isolates from canine otitis in the Iberian Peninsula. The predilection of *P. aeruginosa* for the external ear canal may be attributed to its preference for moist environments and its ability to form biofilms, which are particularly problematic in the confined, warm space of the ear canal [23]. Furthermore, *P. aeruginosa* exhibits intrinsic resistance to many antimicrobials and can rapidly acquire resistance, making infections caused by this bacterium particularly challenging to treat [27].

Our findings also identified *Proteus* spp. (8.2% in pyoderma; 16.5% in otitis externa) and *Streptococcus* spp. (18.8% in pyoderma) as significant pathogens. These results are supported by studies conducted in North China [21], France [29], Spain [28], and Italy [12], which also reported these genera as common isolates in canine dermatological infections. The presence of multiple bacterial species in these infections highlights the complex polymicrobial nature of canine pyoderma and otitis externa, which may have implications for treatment approaches.

Our study revealed high levels of AMR among bacterial isolates from canine pyoderma and otitis externa cases in Hong Kong, with 94% and 93.9% of isolates, respectively, resistant to at least one antimicrobial agent. Resistance was most prevalent against orbifloxacin (61.3% in pyoderma; 76.7% in otitis externa), doxycycline (59.3%; 69.2%), clindamycin (62%; 68.9%), and enrofloxacin (50%; 55.5%). These findings suggest that these antimicrobials may have limited efficacy for empirical treatment of canine pyoderma and otitis externa in Hong Kong. Similar patterns of resistance have been reported in other regions, although with some variations. Huerta et al. [30] found that 78% of staphylococcal isolates from canine pyoderma in Spain were resistant to at least one antimicrobial agent, while Dinkova and Rusenova [31] observed increasing resistance of *Staphylococcus* spp. isolates to amoxicillin/clavulanic acid and cephalexin in Bulgaria from 2022 to 2023. Notably, almost half of the isolates (50% in pyoderma; 55.5% in otitis externa) were resistant to enrofloxacin. In contrast, Tesin et al. [5] found that enrofloxacin showed the lowest resistance against all bacterial isolates recovered from canine otitis externa cases in Serbia, highlighting the importance of regional surveillance data for guiding antimicrobial use. Additionally, our study found that most isolates remained sensitive to ofloxacin, ticarcillin–clavulanate, tobramycin, ciprofloxacin, cefpodoxime, cefuroxime, and cefixime. This finding provides valuable guidance for veterinarians in Hong Kong when selecting antimicrobial therapy for resistant infections. However, it is crucial to emphasize that these antimicrobials should be used judiciously, in exceptional cases, and based on culture and susceptibility testing to preserve their long-term efficacy.

The resistance profiles of *S. pseudintermedius* isolates from pyoderma samples were largely consistent with global trends. Resistance to ampicillin (82.1%) and doxycycline (60.7%) was comparable to previously reported rates [20,32,33,34]. Notably, all isolates in our study were susceptible to erythromycin, which contrasts with previously reported resistance rates of 18.2–60.8% [20,32,35]. Additionally, resistance to trimethoprim–sulfamethoxazole was significantly lower in our cohort (39.3%) compared to earlier reports (64.7–66.2%) [20,36]. These similarities and discrepancies may reflect regional differences in antimicrobial usage patterns, strain epidemiology, or host-related factors such as breed predisposition and prior treatment history.

On the other hand, AMR profiles of *P. aeruginosa* from otitis externa samples posed significant therapeutic challenges. All isolates exhibited resistance to more than three antimicrobials, which is consistent with findings from other regions [5,12,37]. The high levels of resistance observed may be attributed to both the intrinsic and acquired resistance mechanisms of *P. aeruginosa*, which contribute to its clinical persistence. These mechanisms include reduced outer membrane permeability, efflux pump systems, production of antibiotic-inactivating enzymes, and horizontal gene transfer of resistance determinants [38,39]. Furthermore, biofilm formation, which is reported in up to 90.6% of canine isolates, creates a physical barrier that limits antibiotic penetration and contributes to chronic infection [40].

A particularly concerning finding in our study was the high proportion of MDR, detected in 67.5% of pyoderma isolates and 66.8% of otitis externa isolates. Gram-negative bacteria exhibited significantly higher MDR rates than Gram-positive isolates, with the MAR index averaging 0.41 for pyoderma and 0.52 for otitis externa isolates. These results indicate widespread AMR and suggest that a significant proportion of infections may be difficult to treat with conventional antimicrobial therapy. Similar levels of MDR have been reported in other regions. Rosales et al. [23] found MDR in 47% of bacteria isolated from canine otitis in Spain, while Dinkova and Rusenova [31] reported MDR in 38.7% of bacterial isolates from dogs in Bulgaria. Garcias et al. [28] also noted higher MDR rates among Gram-negative species such as *P. mirabilis* (33%) and *E. coli* (25%), which is consistent with our findings. The high proportion of MDR observed in this study may be attributed to several factors, including the widespread use of antimicrobials in veterinary practice, inappropriate dosing or duration of treatment, and the use of broad-spectrum antimicrobials without prior culture and susceptibility testing. Additionally, the close contact between humans and companion animals may facilitate the exchange of resistant bacteria or resistance genes, contributing to the spread of MDR [5].

High levels of MDR *S. pseudintermedius* isolates (53.6%) were observed in the present study, which aligns with previously reported rates ranging from 47% to 62% [33,34,41]. Multiple studies have also demonstrated that MDR *S. pseudintermedius* strains exhibit enhanced biofilm-forming capacity compared to susceptible isolates [41,42], which can compromise treatment efficacy. Ten *S. pseudintermedius* isolates in our study showed resistance to oxacillin, suggesting phenotypic MRSP. This finding is consistent with growing global reports of MRSP in canine pyoderma and otitis externa [43,44,45], reflecting its emergence as a major AMR concern in veterinary medicine. Although methicillin resistance was not specifically assessed in our study, it has been reported in several reference studies. Rosales et al. [23] detected a 26.4% prevalence of MRSP in canine otitis in Spain, while Ludwig et al. [25] reported that 6.3% of *S. pseudintermedius* isolates from European dogs were confirmed *mec*A-positive (methicillin-resistant). The emergence of MRSP is particularly concerning as these strains are typically resistant to all *β*-lactam antimicrobials and often carry additional resistance determinants, limiting treatment options [23].

Other bacterial isolates showing high MDR rates in our study included *P. aeruginosa* (98.2%) and *P. mirabilis* (60.5%). Similar trends have been observed in other studies [28,46,47]. These resistance patterns pose significant therapeutic challenges in managing canine otitis externa, often resulting in treatment failure and recurrent infections, which can further drive the development and spread of AMR through continued selective pressure.

Our findings strongly reinforce the critical importance of prioritizing topical therapy, particularly antiseptics and topical antimicrobial preparations, as the primary treatment modality for canine pyoderma and otitis externa. This approach is in strict accordance with current veterinary dermatology guidelines, which unequivocally advocate for targeted, localized treatment whenever possible to minimize selective pressure for antimicrobial resistance [1]. While common bacterial isolates such as *S. pseudintermedius* and *P. aeruginosa* were frequently associated with both pyoderma and otitis externa in our study, effective topical treatments, coupled with addressing the primary cause, can often resolve these infections without the necessity of systemic antibiotics [19,48]. Therefore, we emphasize that systemic antimicrobial therapy must be used sparingly and reserved exclusively for clinically justified cases, such as deep pyoderma or severe otitis with systemic signs, and should invariably be guided by culture and susceptibility testing, especially in regions with high levels of MDR. This judicious approach not only improves therapeutic outcomes but also significantly supports responsible antimicrobial use and the broader goals of antimicrobial stewardship in veterinary medicine.

Several limitations of our study should be acknowledged. First, as a retrospective analysis, our study was limited to the data available in the laboratory records, which may not have included comprehensive clinical information or follow-up data on treatment outcomes. Second, our study focused on bacterial isolates from clinical cases and did not include samples from healthy dogs, which would have provided valuable comparative data on the normal microbiota and background levels of resistance. Third, although the data were extracted from a single laboratory, they are still representative of the dog population in Hong Kong. Fourth, the distribution of samples across the study period was uneven. Specifically, over half of the pyoderma samples were collected in 2020 and 2022, while more than 60% of otitis externa samples were submitted between 2020 and 2021. This clustering does not necessarily reflect actual disease trends but may instead be influenced by external factors such as changes in submission behavior, diagnostic practices, or data availability during those years. Additionally, our study did not investigate the molecular mechanisms of resistance or the genetic relatedness of isolates, which would have provided insights into the epidemiology and spread of resistant clones. Future studies incorporating whole genome sequencing and molecular typing would enhance our understanding of the dynamics of AMR in canine pathogens.

## 4. Materials and Methods

### 4.1. Study Design and Samples

A retrospective cross-sectional study was conducted to assess the frequency of bacterial infections and their AMR profiles in canine pyoderma and otitis externa cases in Hong Kong between 2018 and 2022. This study analyzed 215 swab samples from canine skin or external ear canals lesions submitted by veterinary clinics to the CityU VDL for bacterial culture and AST.

### 4.2. Data Collection and Management

The Laboratory Information Management System (LIMS) of CityU VDL was searched for canine clinical samples submitted by veterinarians in Hong Kong between October 2018 and December 2022 (Figure 1). Electronic records meeting the following criteria were downloaded: (1) samples from confirmed cases of canine bacterial skin or external ear canal infections and (2) samples submitted for microbiological examination, with or without AST. A total of 215 electronic diagnostic records were retrieved. From each record, the following data were extracted: dog age, sex, breed, date of birth, clinic name, sample type, sample site, sampling date, bacteriological identification results, and AMR profiles. The extracted data were entered into Microsoft Excel for cleaning and assessment of duplicates and missing information.

### 4.3. Microbiological Examination and Identification

Canine skin and ear swabs of suspected pyoderma and otitis externa cases were processed using a standard bacterial culture and identification protocol previously published [49]. Specimens were inoculated onto appropriate aerobic media, and isolated bacteria were identified using matrix-assisted laser desorption/ionization time-of-flight (MALDI-TOF) mass spectrometry (Bruker Daltonic, Bremen, Germany). The MALDI Biotyper software version 3.4 (Bruker Daltonic, Bremen, Germany) was used for data analysis and species-level classification.

### 4.4. Antimicrobial Susceptibility Testing

AST was performed using the disk diffusion method or broth microdilution techniques (using Sensititre system) with 13 antimicrobial classes, including aminoglycosides (gentamicin, tobramycin), amphenicols (chloramphenicol, florfenicol), β-lactams (amoxicillin/clavulanate, ticarcillin–clavulanate, and ampicillin), cephalosporins (cefixime, ceftriaxone, cefuroxime, cefazolin, cefovecin, cefpodoxime, and cephalexin), fluoroquinolones (ciprofloxacin, enrofloxacin, marbofloxacin, ofloxacin, and orbifloxacin), macrolides (erythromycin, azithromycin), tetracyclines (doxycycline, tetracycline), and other antimicrobials (clindamycin, trimethoprim–sulfamethoxazole). The following reference strains were used for internal quality control: *E. coli* ATCC 25922, *P. aeruginosa* ATCC 27853, *S. aureus* ATCC 25923, and *S. pneumoniae* ATCC 49619.

The results were interpreted as sensitive, intermediate, or resistant based on breakpoints from the Clinical and Laboratory Standards Institute (CLSI) VET01S and M100 guidelines applicable in the year of sampling and testing [50,51]. In addition, bacterial isolates exhibiting intrinsic resistance were defined based on the CLSI VET01S and were excluded from the analysis [51].

Antimicrobial resistance rates were reported only for antibiotics with a minimum of 30 tested isolates [50]. The degree of AMR was assessed using the multiple antibiotic resistance (MAR) index, which is calculated as the ratio of the number of antimicrobials to which an isolate was resistant, divided by the total number of antimicrobials tested [52]. Multidrug resistance (MDR) was defined as resistance to ≥1 agent in ≥3 antimicrobial classes [53].

### 4.5. Data Analysis

The data extracted from the laboratory database was organized and curated in Microsoft Excel and then imported into R software (version 4.3.1, R Core Team, Vienna, Austria) for analysis and visualization. The R package “Complex-Heatmap” was used to create a heatmap based on the antimicrobial resistance profiles of each isolate [54]. Normality of the MAR index was assessed using the Shapiro–Wilk test. As the data significantly deviated from normality, the Kruskal–Wallis test was employed to evaluate differences in the index across sampling year, sex, age, sampling site, and bacterial species for both pyoderma and otitis externa cases. When the Kruskal–Wallis test yielded a significant result (*p*-value < 0.05), pairwise comparisons were conducted using Dunn’s post hoc test with Bonferroni correction. Univariable logistic regression analyses were performed to assess the association between MDR (1 = yes, 0 = no) and sampling year, breed, sex, reproductive status, age, sampling site, and bacterial species. A *p*-value ≤ 0.05 was considered statistically significant. Additionally, a geographical map of veterinary clinic locations was generated using QGIS software (version 3.30.3, QGIS Development Team, Zurich, Switzerland).

## 5. Conclusions

Our study provides comprehensive data on the frequency and AMR profiles of bacterial isolates associated with canine pyoderma and otitis externa clinical samples in Hong Kong between 2018 and 2022. *Staphylococcus* spp., particularly *S. pseudintermedius*, were the most frequently isolated, and the high rates of resistance to commonly used antimicrobials highlight the challenges in managing these common canine conditions. The significant prevalence of MDR isolates is particularly concerning and emphasizes the urgent need for prioritized topical antiseptic treatment, and where oral antimicrobial implementation is really needed, routine culture and susceptibility testing should be initiated.

These findings provide critical, region-specific insights that can guide antimicrobial selection where indicated, enhance antimicrobial stewardship efforts, and promote diagnostic-driven treatment strategies to improve clinical outcomes.

From a One Health perspective, the potential for transmission of resistant bacteria between companion animals and humans underscores the importance of a coordinated approach to antimicrobial stewardship that encompasses both veterinary and human medicine. Regular surveillance of AMR in companion animals, along with the development and implementation of evidence-based treatment guidelines, is essential for preserving the efficacy of antimicrobials and ensuring optimal outcomes for both animal and human health.

## Figures and Tables

**Table 1:** Number and distribution of canine pyoderma and otitis externa clinical samples submitted to CityU VDL in Hong Kong over a five-year period.

Characteristics	Number (%) of Dogs with		Total (*n* = 215)
	Pyoderma (*n* = 63)	Otitis Externa (*n* = 152)	
Sampling years			
2018	3 (4.8)	3 (2.0)	6 (2.8)
2019	6 (9.5)	26 (17.1)	32 (14.9)
2020	21 (33.3)	47 (30.9)	68 (31.6)
2021	13 (20.6)	49 (32.2)	62 (28.8)
2022	20 (31.8)	27 (17.8)	47 (21.9)
Breed			
Purebred	59 (93.6)	149 (98.0)	208 (96.7)
Mixed	4 (6.4)	3 (2.0)	7 (3.3)
Sex			
Male	43 (68.2)	82 (54.3)	125 (58.4)
Female	20 (31.8)	69 (45.7)	89 (41.6)
Reproductive Status			
Intact	35 (55.6)	48 (31.6)	83 (38.6)
Desexed	28 (44.4)	104 (68.4)	132 (61.4)
Age (years)			
<2	8 (12.7)	10 (6.6)	18 (8.4)
2 to <5	15 (23.8)	27 (17.7)	42 (19.5)
5 to <10	29 (46.0)	72 (47.4)	101 (47.0)
≥10	11 (17.5)	43 (28.3)	54 (25.1)
Sampling site			
Head	4 (6.4)	--	4 (1.9)
Neck	4 (6.4)	--	4 (1.9)
Trunk	32 (50.7)	--	32 (14.9)
Limbs	16 (25.4)	--	16 (7.4)
Perineal	4 (6.4)	--	4 (1.9)
Tail	3 (4.7)	--	3 (1.4)
Ear (Unilateral)	--	127 (83.6)	127 (59.0)
Ear (Bilateral)	--	25 (16.4)	25 (11.6)
Bacterial infection			
Yes	51 (80.9)	133 (87.5)	184 (85.6)
No	12 (19.1)	19 (12.5)	31 (14.4)

**Table 2:** Number and distribution of antimicrobial resistances in common bacterial isolates recovered from canine pyoderma and otitis externa clinical samples submitted to CityU VDL in Hong Kong over a five-year period.

Bacterial Isolates	No. of Resistances ^1^						MDR ^2^ *n* (%)	Average MAR Index (Range) ^3^
	Total N	0 *n* (%)	1 *n* (%)	2 *n* (%)	3 *n* (%)	>3 *n* (%)		
**I. Pyoderma**								
*Staphylococcus* spp.	35	5 (14.3)	6 (17.1)	3 (8.6)	2 (5.7)	19 (54.3)	15 (42.9)	0.37 (0.05–1.00)
*Streptococcus* spp.	16	--	1 (6.3)	1 (6.3)	1 (6.3)	13 (81.3)	12 (75)	0.32 (0.07–0.57)
*Proteus* spp.	7	--	--	--	1 (14.3)	6 (85.7)	6 (85.7)	0.35 (0.14–0.57)
*Pseudomonas* spp.	4	--	--	--	--	4 (100)	4 (100)	0.76 (0.69–0.88)
**II. Otitis externa**								
*Staphylococcus* spp.	76	13 (17.1)	8 (10.5)	7 (9.2)	5 (6.6)	43 (56.6)	35 (46.1)	0.49 (0.05–1.00)
*Pseudomonas* spp.	56	--	--	--	--	56 (100)	55 (98.2)	0.75 (0.58–0.94)
*Proteus* spp.	38	--	--	--	4 (10.5)	34 (89.5)	23 (60.5)	0.38 (0.18–1.00)
*Corynebacterium* spp.	20	--	3 (15)	1 (4.5)	--	16 (80)	13 (65)	0.42 (0.06–0.82)
*Streptococcus* spp.	14	--	--	--	1 (7.1)	13 (92.9)	9 (64.3)	0.48 (0.17–0.94)
*Escherichia* spp.	13	--	2 (15.4)	2 (15.4)	1 (7.7)	8 (61.5)	7 (53.8)	0.40 (0.06–1.00)
*Enterococcus* spp.	5	1 (20.0)	--	--	--	4 (80.0)	4 (80.0)	0.72 (0.59–0.82)

^1^ N = number of isolates; *n* = number of resistant isolates; ^2^ MDR = multidrug resistance; and ^3^ MAR = multiple antibiotic resistance index.

**Table 3:** Distribution of multidrug-resistance (MDR) in isolates recovered from canine pyoderma and otitis externa clinical samples submitted to CityU VDL in Hong Kong over a five-year period.

Factor		Pyoderma			Otitis Externa		
		MDR (%)	OR (95% CI) ^1^	*p*-Value	MDR (%)	OR (95% CI) ^1^	*p*-Value
Sampling years							
	2018	57.1	Ref.	0.807	66.7	Ref.	0.390
	2019	100	--	--	62.2	0.82 (0.07–9.9)	0.877
	2020	71.4	1.88 (0.34–10.3)	0.470	64.6	0.91 (0.08–10.6)	0.942
	2021	60.0	1.13 (0.18–6.9)	0.899	63.9	0.88 (0.08–10.2)	0.921
	2022	61.5	1.20 (0.30–5.9)	0.833	80.8	2.1 (0.17–25.7)	0.574
Breed							
	Mixed	42.9	Ref.		100	Ref.	
	Purebred	69.7	3.10 (0.64–14.8)	0.162	65.8	--	--
Sex							
	Male	62.5	Ref.		62.6	Ref.	
	Female	69.5	1.37 (0.51–3.7)	0.538	71.4	1.49 (0.85–2.6)	0.160
Reproductive Status							
	Desexed	70.0	Ref.		62.8	Ref.	
	Intact	66.0	0.83 (0.32–2.2)	0.711	75.0	1.78 (0.96–3.3)	0.065
Age (years)							
	<2	66.7	Ref.	0.993	60.0	Ref.	0.599
	2 to <5	65.2	0.94 (0.18–48)	0.938	72.2	1.73 (0.49–6.1)	0.394
	5 to <10	68.4	1.08 (0.23–5.1)	0.919	69.0	1.48 (0.50–4.5)	0.486
	≥10	69.2	1.13 (0.18–6.9)	0.899	61.3	1.06 (0.33–3.3)	0.927
Sampling site							
	Head	80.0	Ref.	0.675	--	--	--
	Neck	100.0	--	--	--	--	--
	Trunk	59.5	0.37 (0.04–3.6)	0.389	--	--	--
	Limbs	70.4	0.59 (0.06–6.2)	0.663	--	--	--
	Perineal	100.0	--	--	--	--	--
	Tail	50.0	0.25 (0.01–8.6)	0.442	--	--	--
	Ear (Unilateral)	--	--	--	66.8	Ref.	
	Ear (Bilateral)	--	--	--	66.7	0.99 (0.45–2.3)	0.986
Bacterial species							
	Gram-positive	56.1	Ref.		53.1	Ref.	
	Gram-negative	92.3	9.4 (2.0–43.5)	0.004	80.7	3.70 (2.0–6.7)	<0.001

^1^ OR = odds ratio; CI = confidence interval.

## Data Availability

The data presented in this study are available upon request from the corresponding author.

## Supplementary Materials

The following supporting information can be downloaded at https://www.mdpi.com/article/10.3390/antibiotics14070685/s1: Figure S1: Heatmap for antimicrobial resistance for the most prevalent bacterial species isolated from canine pyoderma and otitis externa clinical samples in Hong Kong between 2018 and 2022.

## Abbreviations

AMR	Antimicrobial resistance
MDR	Multidrug resistance
MAR	Multiple antibiotic resistance index
MRS	Methicillin-resistant staphylococcus
