# Supplementary material for: Canine Pyoderma and Otitis Externa: A Retrospective Analysis of Multidrug-Resistant Bacterial Carriage in Hong Kong
Source: Antibiotics (Basel). 2025 Jul 6;14(7):685. doi: 10.3390/antibiotics14070685 (PMC12291705; doi:10.3390/antibiotics14070685)

Figure S1. Heatmap for antimicrobial resistance for most prevalent bacterial species isolated from canine pyoderma and otitis externa clinical samples in Hong Kong between 2018 and 2022.

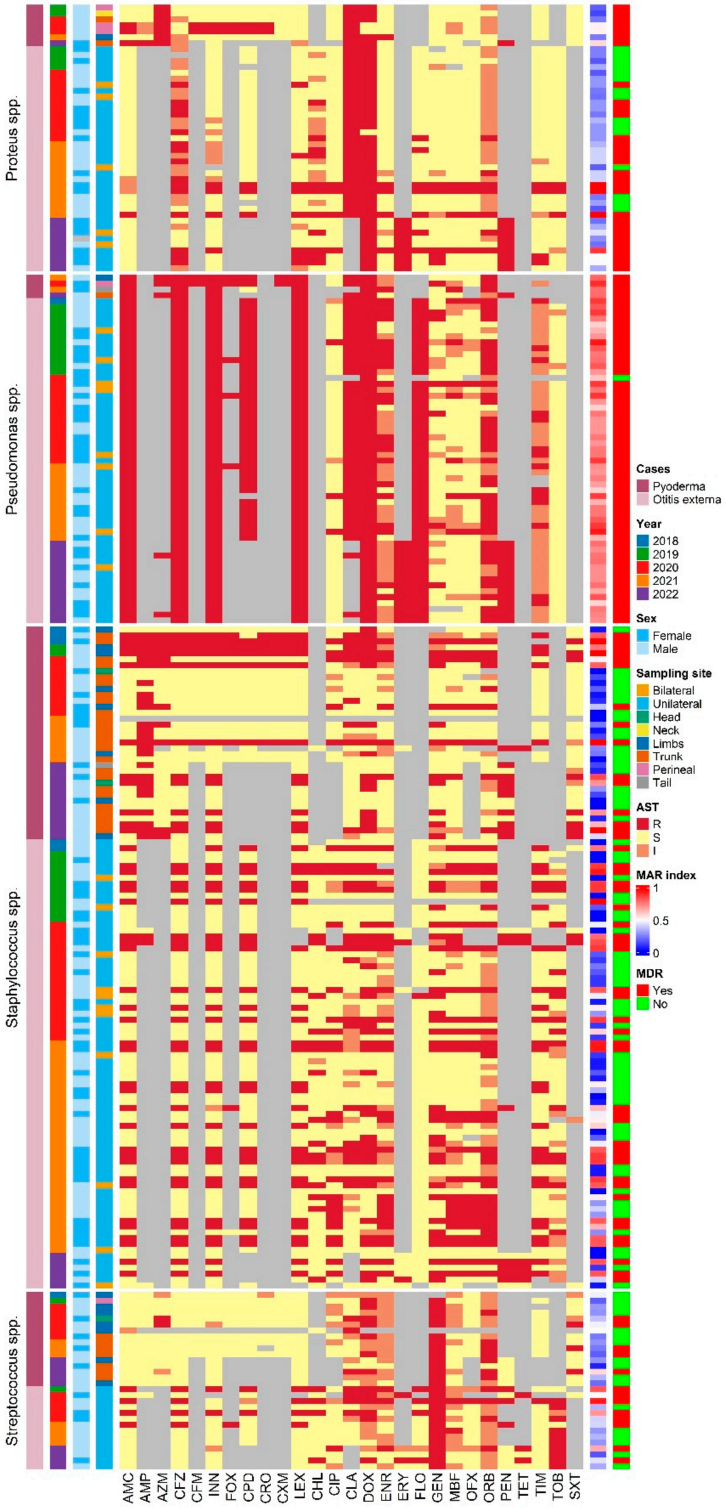

Supplement: Supplementary file 1 [file antibiotics-14-00685-s001.zip › antibiotics-3719907-supplementary.pdf]
